# Proteomic Analysis of Rice Subjected to Low Light Stress and Overexpression of *OsGAPB* Increases the Stress Tolerance

**DOI:** 10.1186/s12284-020-00390-8

**Published:** 2020-06-01

**Authors:** Yangxuan Liu, Ting Pan, Yuying Tang, Yong Zhuang, Zhijian Liu, Penghui Li, Hui Li, Weizao Huang, Shengbin Tu, Guangjun Ren, Tao Wang, Songhu Wang

**Affiliations:** 1 Chengdu Institute of Biology, Chinese Academy of Sciences, Chengdu, 610041 China; 2 University of Chinese Academy of Sciences, Beijing, 100049 China; 3 Southeast Asia Biodiversity Research Institute, Chinese Academy of Sciences, Yezin, Nay Pyi Taw, 05282 Myanmar; 4 Crop Research Institute, Sichuan Academy of Agricultural Sciences, Chengdu, 610066 People’s Republic of China; 5 School of Horticulture, Anhui Agricultural University, Hefei, 230036 China

## Abstract

**Background:**

Light provides the energy for photosynthesis and determines plant morphogenesis and development. Low light compromises photosynthetic efficiency and leads to crop yield loss. It remains unknown how rice responds to low light stress at a proteomic level.

**Results:**

In this study, the quantitative proteomic analysis with isobaric tags for relative and absolute quantitation (iTRAQ) was used and 1221 differentially expressed proteins (DEPs) were identified from wild type rice plants grown in control or low light condition (17% light intensity of control), respectively. Bioinformatic analysis of DEPs indicated low light remarkably affects the abundance of chloroplastic proteins. Specifically, the proteins involved in carbon fixation (Calvin cycle), electron transport, and ATPase complex are severely downregulated under low light. Furthermore, overexpression of the downregulated gene encoding rice β subunit of glyceraldehyde-3-phosphate dehydrogenase (OsGAPB), an enzyme in Calvin cycle, significantly increased the CO_2_ assimilation rate, chlorophyll content and fresh weight under low light conditions but have no obvious effect on rice growth and development under control light.

**Conclusion:**

Our results revealed that low light stress on vegetative stage of rice inhibits photosynthesis possibly by decreasing the photosynthetic proteins and *OsGAPB* gene is a good candidate for manipulating rice tolerance to low light stress.

## Background

In the natural environment, the sessile plants must respond to fluctuations of sunlight which provide the energy for photosynthesis and determines the plant morphogenesis and development (Ruberti et al. 2012; Vialet-Chabrand et al. 2017). Low light is considered as abiotic stress that compromises photosynthesis and crop yield potential (Tian et al. 2017; Kaiser et al. 2018). Morphologically, low light affects plant height, biomass, and root growth (Liu and Su 2016). In rice growth, low light reduces tillering, panicle and spikelet numbers, and grain weight and quality (Sun et al. 2012; Wang et al. 2013; Sekhar et al. 2019). In some areas with continuously cloudy weather or rainfall, rice yields can be reduced by 30% to 50% (Venkateswarlu 1977; Viji et al. 1997; Liu et al. 2009), indicating that low light is an indispensable problem for rice production. A global rice diversity survey of biomass accumulation revealed that the photosynthetic rate under low light is highly related to biomass accumulation and it has great potential to be used as a target for rice high yield breeding (Qu et al. 2017).

The lower activities of photosystem (PS) II, ATP synthase, cytochrome (Cyt) b/f, and ribulose-1,5-bisphosphate carboxylase/oxygenase (Rubisco), electron transport (ETR), and CO_2_ consumption were observed in the plants under low light (Leong and Anderson 1984; Zivcak et al. 2014). In addition, low light stress causes oxidative damage and D1 protein degradation by generation of active oxygen species (Keren et al. 1997). Also, low light stress induces PS state transitions (State 1 - State 2 transitions), which maximize the efficiency of light-harvesting at low light intensity (Mullineaux and Emlyn-Jones 2005; Tikkanen et al. 2006).

Manipulation of chloroplastic genes can promote plant acclimation to low light stress. Transgenic plants with a decreased Transkelolase activity showed the enhanced tolerance to low temperature and weak light stress (Bi et al. 2015). Overexpression of the Rubisco activase increased the tolerance to low light in cucumber (Bi et al. 2017). Overexpression of zeaxanthin epoxidase gene from *Medicago sativa* also improve the tolerance to low light stress in tobacco (Cao et al. 2018). In addition, the exogenous application of phytohormones is a practical way to increase low light tolerance. 24-Epibrassinoslide, one of the active and stable forms of Brassinosteroids, elicit synergism between the antioxidant activities and ATP synthase β subunit and promote tomato tolerance to low temperature and low light stress (Cui et al. 2016). The foliage spray of GR24, a synthesized strigolactone, alleviated photosynthetic inhibition and oxidative stress of tomato seedlings under low light stress (Lu et al. 2019). Interestingly, the appropriate ratio of NH_4_^+^:NO_3_^−^ improves low light tolerance in mini Chinese cabbage seedlings (Hu et al. 2017).

Analysis of rice varieties tolerant to low light indicated that higher chlorophyll content, higher efficiency in photosynthesis, and stronger antioxidant ability improve plant tolerance to low light stress (Nayak and Murty 1980; Liu et al. 2014). A comparative transcriptome analysis indicated that those genes encoding some of the subunits of the light-harvesting complex, PS I and II complex are up-regulated in low light-tolerant rice variety (Swarnaprabha) under low light condition compared with the low light-sensitive one (IR8) (Sekhar et al. 2019). In addition, carbohydrate level and flux are also important because the altered expression of malate-permeable anion channel *OsALMT4* reduces the growth of rice under low light (Liu et al. 2018).

Although the alterations of the rice transcriptome in response to low light have been investigated, the proteome analysis is still lacking. In this study, we used quantitative proteomic analysis with isobaric tags for relative and absolute quantitation (iTRAQ) to investigate the protein level alterations in rice acclimation to low light.

## Results

### Quantitative Proteomic Analysis with iTRAQ

**Fig. 1:**
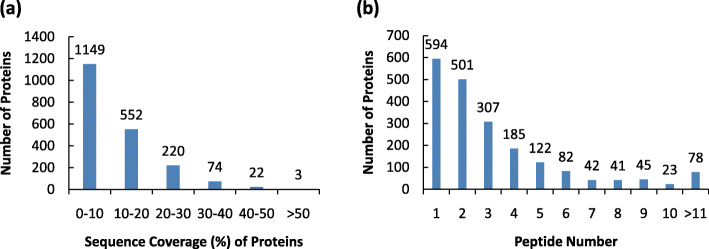
Summary of iTRAQ proteomic data. **a** Sequence coverage (%) of proteins by the identified peptides. The bars were labeled by the number of identified proteins distributed in different ranges of sequence coverage; **b** Graphical representation of the distribution of the identified peptide number for the proteins

The 2-week-old seedlings of wild type rice Nippobare (NIP) were treated under low light condition (17% control light) for 5 days. Total protein extraction and iTRAQ-based proteome were performed in the company BASEBIO (see details in methods). Using the criteria of Score Sequest HT > 0 and unique peptides ≥1, total 17,192 peptides were identified by the tandem mass spectrometry (MS/MS) (Table 1). Among them, 5697 unique peptides and 2020 majority proteins were characterized (Table 1). The related information of detected peptide and protein groups were list in Supplemental Table 1 and Supplemental Table 2, respectively. About 43% of the identified majority proteins have more than 10% peptide sequence coverage (Fig. 1a). And approximately 70% of the identified majority proteins have more than 2 peptide hits (Fig. 1b).

**Table 1:** The statistics information of the peptides identified by iTRAQ proteomic analysis

Category	Peptide Number
Total MS/MS Count	17192
Unique Peptides	5697
Majority protein IDs	2020
Deamidation site IDs	202
Oxidation site IDs	294
Low_VS_CK: differentially expressed proteins	1221
Low_VS_CK: upregulated proteins	223
Low_VS_CK: downregulated proteins	998

### Functional Analysis of Differentially Expressed Proteins (DEPs)

To further explore how rice responds to low light stress, proteins with fold change (FC) > 1.2 and *p*-value< 0.05 were considered as upregulated, while those with FC < 5/6 and p-value< 0.05 were considered as downregulated. These cutoffs were chosen according to a previous iTRAQ proteomic analysis on rice (Xiong et al. 2019). Comparison between CL and LL revealed 1221 DEPs including 223 upregulated and 998 downregulated proteins (Table 1 and Supplemental Table 3). Our results indicated that more than 80% of DEPs were downregulated.

**Fig. 2:**
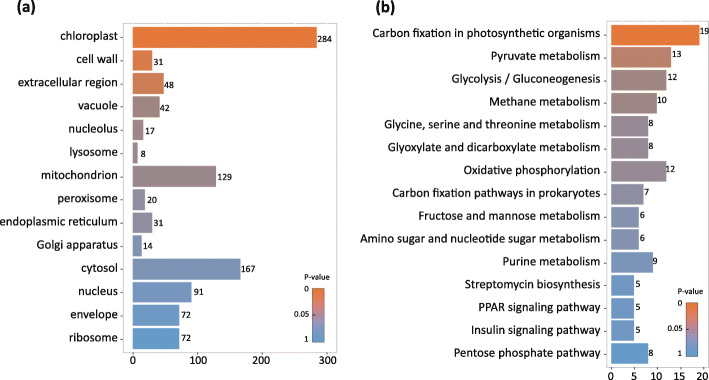
GO and KEGG analysis of differentially-expressed proteins under control and low light conditions. **a** GO enrichment analysis of all differentially expressed proteins on cellular component; **b** KEGG pathway enrichment analysis of differentially expressed proteins

According to the subcellular compartment categories, gene ontology (GO) analysis indicated that the identified DEPs cover all the subcellular organelles (Fig. 2a and Supplemental Table 4). Low light stress significantly affects the proteins from all three organelles including chloroplast, cell wall, and extracellular region (Fig. 2a). Among them, the chloroplast is the most severely-affected organelle, as indicated by that 284 chloroplastic proteins were differently accumulated under low light stress (Fig. 2a). In addition, 31 and 48 DEPs were detected in cell wall and extracellular region, respectively (Fig. 2a). Although mitochondria and cytosol contain many DEPs, they are not significant because of *P*-value> 0.05 (Fig. 2a).

To analyze the metabolism response to low light stress, DEPs were further classified by Kyoto encyclopeida of genes and genomes (KEGG) database. The results show that DEPs were majorly enriched in carbon metabolism, such as carbon fixation in photosynthetic organisms, pyruvate metabolism, and glycolysis/gluconeogenesis (Fig. 2b and Supplemental Table 5). Carbon fixation in photosynthetic organisms and pyruvate metabolism, containing 19 and 13 DEPs respectively, were significantly affected under low light stress (Fig. 2b).

### Proteins Involved in Photosynthesis are Downregulated

**Fig. 3:**
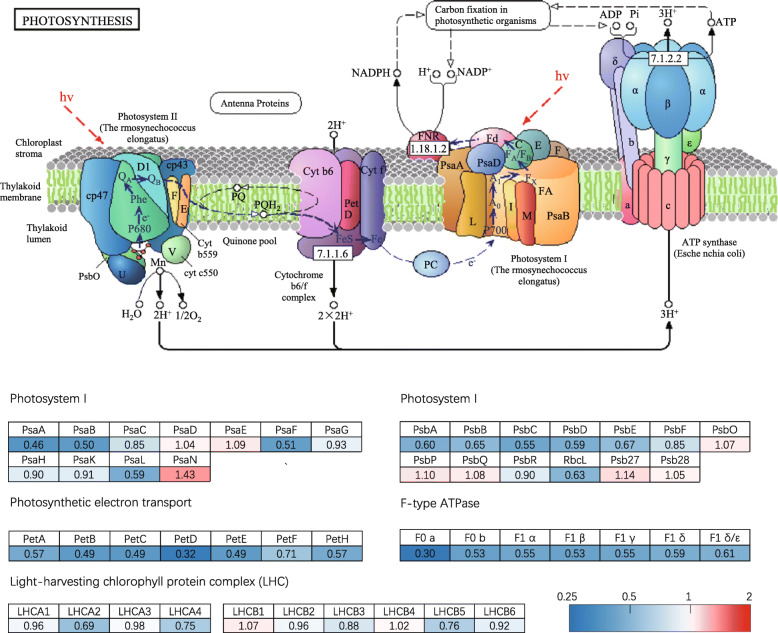
Low light alters the abundance of proteins involved in photosystems. The detected subunits of Photosystem I, II, photosynthetic electron transport, F-type ATPase, and Light-harvesting chlorophyll protein complex were labeled with fold changes after low light stress. The blue indicates the downregulation while the red indicates the upregulation

**Fig. 4:**
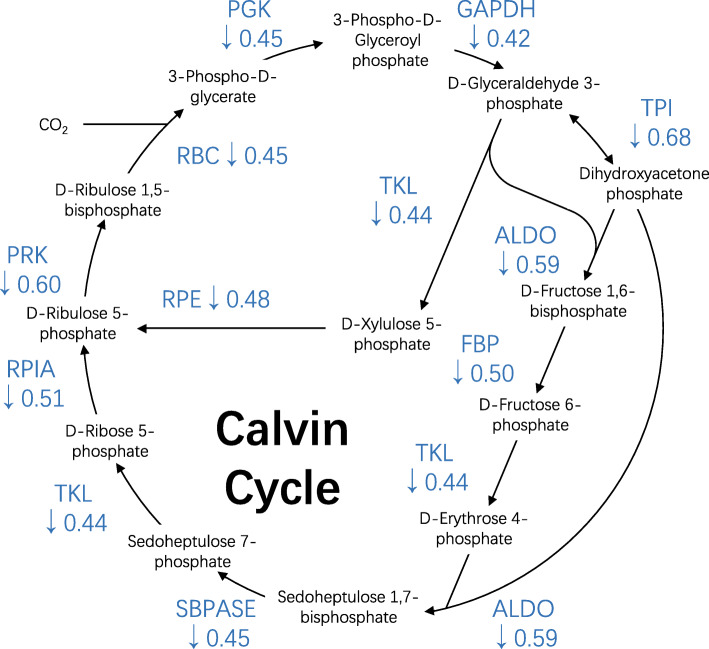
Low light suppresses the Calvin cycle. The detected proteins involved in Calvin cycle were labeled by the fold changes after low light stress. The “downward arrow” indicates the downregulation. ALDO, fructose-bisphosphate aldolase; TKL, transketolase; SBPASE, sedoheptulose-bisphosphatase; FBP, fructose-1,6-bisphosphatase; GAPDH, glyceraldehyde 3-phosphate dehydrogenase (phosphorylating); PGK, phosphoglycerate kinase; PRK, phosphoribulokinase; RBC, ribulose-bisphosphate carboxylase; RPE, ribulose-phosphate 3-epimerase; RPIA, ribose 5-phosphate isomerase A; TPI, triosephosphate isomerase

Since low light stress significantly influences the chloroplast proteome, we further analyzed the proteins involved in photosystems and Calvin Cycle in detail. As shown in Fig. 3, some subunits of PSI and PSII complexes are downregulated after low light treatment. Besides, all the detected proteins involved in photosynthetic electron transport and F-type ATPase complex are downregulated (Fig. 3). Relatively, the light-harvesting chlorophyll complex is less affected and only three subunits (LHCA2, LHCA4, and LHCB5) are significantly downregulated (Fig. 3). Calvin cycle is responsible for photosynthetic carbon fixation in chloroplasts. The catalytic enzymes in this pathway are all detected in our proteomic analysis (Fig. 4). The protein levels of these enzymes are all remarkably decreased after low light treatment (Fig. 4). Especially, the protein level of glyceraldehyde-3-phosphate dehydrogenase (GAPDH) under low light is reduced to 42% of that under control light (Fig. 4). These results suggested that low light stress significantly inhibits photosynthesis, possibly by reducing the abundance of proteins involved in Calvin cycle, electron transport, and F-type ATPase complex in rice.

### Overexpression of *GAPB* Increases Rice Tolerance to Low Light Stress

**Fig. 5:**
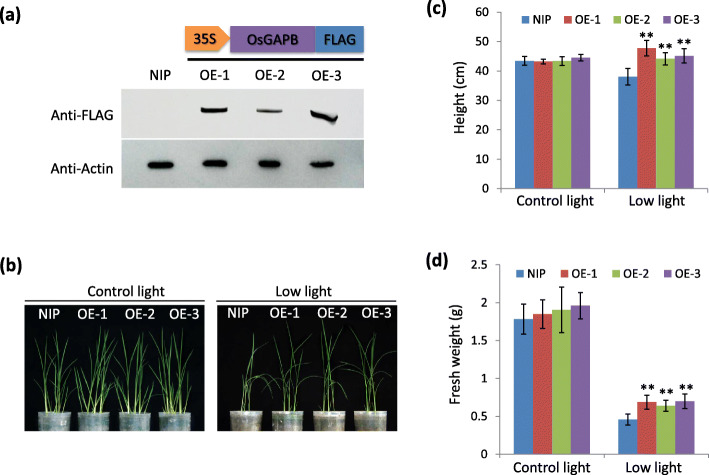
*OsGAPB* overexpression confers the tolerance to low light stress in rice. **a** Immunoblotting analysis of OsGAPB-FLAG protein expression in 35S:OsGAPB-FLAG transgenic (overexpression, OE) lines; **b**, **c**, and **d** The phenotypes **(b)**, height **(c)** and fresh weight **(d)** of 35S:OsGAPB-FLAG transgenic plants under common and low light. The mean ± s.d. values (*n* = 24; “**” indicates *P* < 0.01, two-tailed *t*-test) were calculated from the results of three independent experiments

The previous studies indicated that GAPDH functions are affected by light availability (Fermani et al. 2007; Howard et al. 2008) and β subunit of GAPDH (GAPB) contributes to plant tolerance to abiotic stress in *Arabidopsis* (Chang et al. 2015). Considering our result that low light significantly inhibits GAPDH accumulation (Fig. 4), we attempted to investigate whether *GAPB* overexpression influences rice tolerance to low light stress. The rice *GAPB* (*OsGAPB*) gene (LOC_Os03g03720) was fused with a fragment encoding FLAG tag and the fusion gene *GAPB-FLAG* was driven by the constitutively expressed promoter CaMV35S (Fig. 5a). The construct 35S::GAPB-FLAG was introduced to the wild type rice NIP by agrobacterium-mediated transformation. Three independent transgenic lines, named overexpression (OE)-1, − 2, and − 3, were selected according to the immunoblot analysis with anti-FLAG antibodies which showed that GAPB-FLAG was substantially expressed in these lines (Fig. 5a). The one-week-old plants of T3 generation were grown in soil under control and low light condition, respectively, for 3 weeks (Fig. 5b). The plants of NIP and OE lines showed no obvious difference on plant height (Fig. 5c) and fresh weight (Fig. 5d) under control light. Under low light, however, plant height and fresh weight of OE lines were significantly increased compared to NIP (Fig. 5c and d). These results indicated that *GAPB* overexpression promotes plant tolerance to low light stress.

### GAPB Enhances CO_2_ Assimilation and Chlorophyll Accumulation under Low Light

**Fig. 6:**
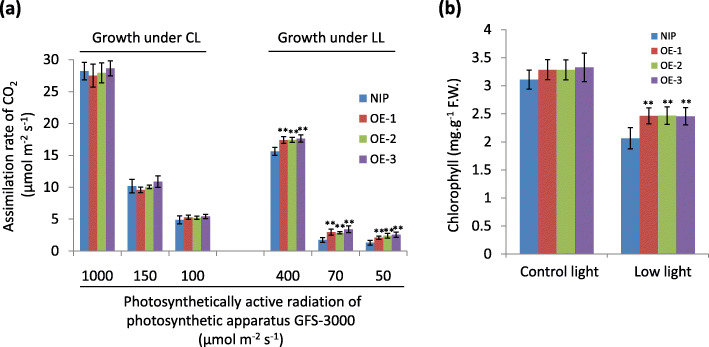
*OsGAPB* overexpression leads to higher CO_2_ asssimilation rate and chlorophyll content. **a** The CO_2_ assimilation rate of NIP and *OsGAPB* overexpression (OE) plants with or without low light treatment. The CO_2_ assimilation rates were measured under different light intensities that were provided by the photosynthetic apparatus GFS-3000; The mean ± s.d. values (*n* = 12; “**” indicates *P* < 0.01, two-tailed *t*-test) were calculated from the results of three independent experiments. **b** Chlorophyll contents of *OsGAPB* OE plants grown under different light conditions; The mean ± s.d. values (*n* = 9; “**” indicates *P* < 0.01, two-tailed *t*-test) were calculated from the results of three independent experiments

To elucidate the reason that *GAPB* overexpression increases fresh weight under low light, we measured the assimilation rate of CO_2_ in NIP and OE plants grown under control and low light, respectively. Our results indicated that *GAPB* overexpression has no obvious impact on CO_2_ assimilation when plants are grown under control light (Fig. 6a). However, OE plants showed higher CO_2_ assimilation rates than NIP plants when subjected to low light stress (Fig. 6a). Meanwhile, the quantitative examination of the chlorophyll contents indicated that *GAPB* overexpression enhances chlorophyll accumulation under low light stress but not under control light (Fig. 6b). These results suggested that GAPB increases low light stress tolerance possibly by enhancing chlorophyll accumulation and photosynthetic rate.

## Discussion

The proteomics approaches have been extensively used to study rice responses to the abiotic stresses (Singh and Jwa 2013) including extreme temperatures (Cui et al. 2005; Lee et al. 2007), drought (Salekdeh et al. 2002), salt stress (Abbasi and Komatsu 2004), heavy metals (Hajduch et al. 2001), UV radiation (Du et al. 2011), and ozone (Agarwal et al. 2002), but not low light stress. The systematic comparison of these proteomic studies indicated that, at the proteomic level, the most common responses to abiotic stresses involve alterations on photosynthesis apparatus, redox homeostasis, antioxidation pathway, carbohydrate metabolism, and protein metabolism (Singh and Jwa 2013). Our proteomics analysis of low light stress revealed that carbohydrate metabolism (Figs. 2 and 4) and photosynthesis apparatus (Fig. 3) are significantly affected in rice subjected to low light stress, while redox homeostasis, antioxidation pathway, and protein metabolism showed no obvious alteration.

Chloroplast, an organelle responsible for photosynthesis, is most severely affected by low light stress (Fig. 2). The previous studies indicated that low light stress suppresses photosystem (PS) II, ATP synthase, cytochrome (Cyt) b/f, and ribulose-1,5-bisphosphate carboxylase/oxygenase (Rubisco), electron transport (ETR), and CO_2_ assimilation (Leong and Anderson 1984; Zivcak et al. 2014). Some observations are confirmed by our proteomic results that all subunits of ATP synthase, electron transport, and Calvin cycle are substantially decreased under low light (Figs. 3 and 4). PSI and PSII are partially inhibited but the inhibition on the light-harvesting chlorophyll complexes is much less (Fig. 3). D1 (PsbA) protein is also remarkably decreased under low light condition (Fig. 3), which is consistent with the previous study showing that low light stress led to D1 protein degradation (Keren et al. 1997). These results indicated that low light stress inhibits photosynthesis, possibly by reducing the abundance of photosynthetic proteins, especially those involved in Calvin cycle, electron transport, and ATPase complex in rice. Interestingly, cell wall and extracellular region proteins are also significantly affected (Fig. 2), although further investigations are required for elucidating the roles of cell wall and extracellular proteins in response to low light stress.

Calvin cycle of CO_2_ assimilation into carbohydrates is a major pathway for chemical utilization of light energy in all photosynthetic eukaryotes. Calvin cycle enzymes are activated in the light and deactivated in the dark by a thioredoxin-dependent regulation (Buchanan and Balmer 2005; Trost et al. 2006). Our results revealed that low light availability also decreases the protein abundance of Calvin cycle enzymes (Fig. 4) although the underlying mechanisms remain unknown so far. A Calvin cycle multiple-protein complex including phosphoribulokinase (PRK), GAPDH, and a small protein, CP12, has been identified to play a crucial role in the modulation of carbon fixation in response to alterations in the availability of light (Trost et al. 2006; Fermani et al. 2007; Howard et al. 2008). Dark conditions promote the formation of PRK/GAPDH/CP12 complex and the activity of PRK and GAPDH is very low. Light or illumination induces dissociation of the supramolecular complex, which is accompanied by activation of PRK and GAPDH (Howard et al. 2008). The light-dependent on-off switch of the PRK/GAPDH/CP12 complex can facilitate the coordination of PRK and GAPDH activity in response to changes in light intensity. In our study, PRK and GAPDH are both down-regulated under low light treatment (Fig. 4), suggesting that light intensity also affects the protein abundance of PRK and GAPDH, in addition to post-translational modification and complex formation.

GAPDH has two subunits including GAPA and GAPB and the major GAPDH isoform of land plants is A_2_B_2_-GAPDH. Light-activation of A_2_B_2_-GAPDH depends on the redox state of the C-terminal extension of GAPB (Fermani et al. 2007). Our results showed that overexpression of *OsGAPB* gene increases CO_2_ assimilation rate in rice subjected to low light stress (Fig. 6a), suggesting *OsGAPB* plays an important role in plant acclimation to low light stress. As for the reason, we have several speculations. First is that *GAPB* overexpression increases the GAPDH activities under low light because single GAPB has GAPDH activities when they are purified from *E. coli* although its activity is lower than GAPA (Baalmann et al. 1996). The second is that *GAPB* overexpression possibly leads to the ratio imbalance between GAPA and GAPB, which might disrupt the formation of PRK/GAPDH/CP12 complex that inhibits GAPDH and PRK activity. The next is that GAPB plays roles in maintaining photosynthesis and plant development under salt stress, as indicated by the previous study on *Thellungiella halophila*, a plant surviving from high saline condition (Chang et al. 2015). Interestingly, the chlorophyll contents of *OsGAPB* OE plants are also higher than that of NIP plants (Fig. 6b). The higher chlorophyll contents might also contribute to higher CO_2_ assimilation rate under low light condition. Of course, further studies are required to elucidate how *OsGAPB* overexpression affects CO_2_ assimilation rate and chlorophyll accumulation under low light.

## Conclusion

Our work revealed that low light stress severely inhibits carbon fixation pathway and *OsGAPB* overexpression can increase rice tolerance to low light stress, possibly by enhancing CO_2_ assimilation and chlorophyll accumulation.

## Materials and Methods

### Plant Materials

*Oryza sativa spp. japonica* cv Nipponbare (NIP) was used for proteomic analysis and genetic transformation in this study. The *OsGAPB* overexpression plants were obtained by the following procedures. The *OsGAPB* (LOC_Os03g03720) gene was amplified from total RNAs extracted from mature leaf of NIP by RT-PCR. The 3′ terminus of the *OsGAPB* coding region was fused with a FLAG× 3 tag. After double digestion and ligation reaction, the fusion *OsGAPB-FLAG* gene was cloned into pHB vector (Zhang et al. 2015). Agrobacterium-mediated transformation was performed by a company (Wuhan Doublehelix Biology Science and Technology, China). Three independent transgenic lines (OE-1, − 2, and − 3) were selected and validated by immunoblotting analysis. T3 generation plants were used for phenotypic analysis. The primers for plasmid construction are listed in Supplement Table 6.

### Low Light Treatment and Photosynthesis Rate

After germination in water for 1 week, rice seedlings (NIP) were planted in 2.4 L plate filled with 2.25 kg nutrient soils and fertilized with 0.1 g urea and 0.1 g compound fertilizer containing (15%N, 15%P_2_O_5_, and 15%K_2_O). Rice plants were cultured in a growth chamber equipped with Philips GreenPower LED toplighting module providing common light (300 μmol m^− 2^ s^− 1^) and a photoperiod of 14 h/10 h (day/night). Temperature was controlled by air conditioning at 28 ± 2 °C. After growing in this chamber for 1 week, half of the seedlings were transferred to another chamber, where toplighting was blocked by black nets, with the same temperature and photoperiod but low light (50 μmol m^− 2^ s^− 1^) for another 5 days. Twenty seedlings for each treatment were harvested and frozen in liquid N2 for protein extraction and iTRAQ proteomic analysis.

For pheotypic analysis of the tolerance to low light stress, NIP and OsGAPB overexpression (OE) plants were germinated and grown in the same conditions as described above. Photographs and relevant data were collected after treated in different light conditions for 3 weeks.

The data of photosynthesis was obtained with GFS-3000 (WALZ, German) and its software, through which the assimilation rate was automatically calculated according to a previous study (von Caemmerer and Farquhar 1981). The cuvette temperature (28 °C), flow rate (750 μmol/s), impeller (7), CO_2_ control (off), and RH (50%) were kept constant throughout the measurement. Light provided with GFS-3000 and set as PAR top mode at 1000, 400, 150, 30 μmol/m^2^/s. After warm-up period for about 30 min, calibration was performed with Mode ZP and ZPcuv was stored until the value of dCO_2_ and dH_2_O were stable. Each plant was recorded three readings until the values are stable after about 2 min.

### Protein Preparation

The protein preparation and iTRAQ proteomic analysis were accomplished by Chengdu Basebio Technologies, Inc. Plant samples were washed twice by chilled PBS buffer (Hyclone) and cut into pieces on ice. Two volume Lysis buffer (100 mM Tris-HCl, 4% SDS, pH 8.5) of samples were added to extracted proteins by simple homogenizer and ultrasonic cell disruptor on ice. After boiling at 95 °C for 10 min, the mixture was centrifugated at 4 °C, 30,000 g for 10 min. The supernatant was transported to a new tube and assayed by BCA Protein Assay Kit (Beyotime). 10 mM DTT (final concentration) was added to 200 μg total protein of each sample solution and incubated at 56 °C for 1 h to reduce disulfide bonds in proteins of the supernatant. The solution was transferred to 10 K ultrafiltration tube and centrifuged. After the collection solution was discarded, the precipitate was washed by 200 μL 8 M UA and centrifugated. Subsequently, 200 μL 50 mM IAA was added to the precipitate to block the cysteines and incubated for 1 h in the darkroom. After the precipitate was washed by 200 μL 8 M UA twice, it was dissolved in 200 μL 50 mM TEAB (Applied Biosystems, Milan, Italy) and centrifuged twice. And then the protein was digested with Trypsin Gold (Promega, Madison, WI, USA) with the ratio of protein: trypsin =50: 1 at 37 °C for 16 h.

### iTRAQ Labeling and HPLC Fractionation

After trypsin digestion, peptides were dried by vacuum centrifugation. Peptides were reconstituted in isopropanol and processed according to the manufacture’s protocol for iTRAQ reagent (Applied Biosystems). Briefly, one unit of iTRAQ reagent was thawed and reconstituted in 50 μL isopropanol. Samples were labeled with the iTRAQ tags as follows: Plants treated under low light (0-NIP-LL tag); Plants treated under common light (1-NIP-CK tag). The peptides were labeled with the isobaric tags, incubated at room temperature for 2 h. After incubated with ddH_2_O for 15 min to block labeling, the labeled peptide mixtures were then pooled and dried by vacuum centrifugation.

High performance liquid chromatography was performed for peptide purification. The iTRAQ-labeled peptide mixtures were reconstituted with 0.2% formic acid (FA) and loaded onto a C18 column. The peptides were eluted at a flow rate of 1 mL/min with 95% buffer A (2% ACN in ammonium hydroxide, pH 10) and 5% buffer B (90% ACN in ammonium hydroxide, pH 10) for 5 min, a gradient of 95–60% buffer A and 5–40% buffer B for 30 min, and a gradient of 60–10% buffer A and 40–90% buffer B for 2 min. The system was then maintained at 10% buffer A and 90% buffer B for 2 min before equilibrating with 95% buffer A and 5% buffer B for 5 min before the next injection. Fractions were collected every 1 min. The eluted peptides were pooled into 60 fractions and vacuum-dried.

After reconstituted with 0.1% formic acid (FA), samples were desalted with Oasisi HLB cartridges. Briefly, the cartridges were previously equilibrated by 100% ACN and washed twice by 0.2% FA. After twice injection of samples, the C18 column combined with peptides was washed by 0.1%FA/5% ACN twice. The peptides were eventually eluted by 500 μL 0.1% FA/70% ACN and vacuum-dried.

### HPLC-MS/MS Analysis

The HPLC-MS/MS analysis based on Thermo Fisher Easy-nLC 1000 (Thermo Scientific, San Jose, CA, USA) and Thermo Fisher Q Exactive (Thermo Scientific, San Jose, CA, USA). Each fraction was re-suspended in buffer A (0.1%FA) and was loaded on a 7.5 × 250 mm C18 column containing 3 μm particles. Then the 15 min gradient was run at 300 nL/min starting from 6 to 9% B (100%ACN) for 15 min, followed by 20 min linear gradient to 14%, then, followed by 60 min linear gradient to 30%, followed by 15 min linear gradient to 40%, followed by 3 min linear gradient to 85%, and maintenance at 85% B for 7 min.

Data acquisition was performed with FTMS analyzer with normal mass range, resolution of 70,000, full scan type, positive polarity and data type of profile. The MSn settings was operated with n of 2, Act-Type of HCD, Iso-width (m/z) of 2.0, Normalized-collision-energy of 35.0, Act-Q of 0.250 and Act-Times (ms) of 10. The scan range was applied with first mass (m/z) of 350 and last mass (m/z) of 1800.

### Data Analysis

The identification and quantitation of raw data from HPLC-MS/MS were accomplished by Chengdu Basebio Technologies, Inc. Proteins identification were performed by using MaxQuant (https://www.maxquant.org/; version1.6.1.0) against uniprot-taxonomy Rice. For protein identification, the input data was searched with Enzyme Name of Trypsin (Full), Max-Missed Cleavage Sites of 2, Precursor Mass Tolerance of 20 ppm and Fragment Mass Tolerance of 4.5 ppm. Carbamidomethyl (C) was set as Static Modification, and Oxidation (M) and Deamidated (N,Q) were set as Dynamic Modification. The validation was based on q-value.

### Bioinformatics Analysis

Different expressed proteins were considered as significant only when *p*-values < 0.05 and fold changes > 1.2 or < 5/6 (0.83). The retrieved proteins sequences from Mascot were searched through NCBI BLAST online against non-redundant protein sequences (nr) database (2018.8.22). The BLAST results, which contained the top 20 blast hits with 1 e^− 3^ E-value for each sequence, were loaded into Blast2GO Basic (BioBam, Spain, Version 5.1.13) for Gene Ontology (GO) mapping and annotation using Gene Ontology file go-basic.obo (2018.6). The default annotation configuration was fixed with a GO weight of 5 and annotation cutoff of 75. Un-annotated proteins with BLAST hits were then annotated again with annotation cutoff of 45. All un-annotated proteins’ sequences were then collected to retrieve InterProScan GO functional annotations through InterProScan4 against EBI databases. These annotation data were loaded to Cytoscape (Version 3.6.0), and GO enrichment analysis was accomplished through app BiNGO (Version 3.0.3) within this software. Following the GO enrichment analysis, these studied proteins were blasted against Kyoto Encyclopedia of Genes and Genomes (KEGG) GENES to retrieve the KEGG Orthology (KO) annotation and were subsequently mapped to KEGG pathways through BlastKOALA (https://www.kegg.jp/blastkoala/) online. The KO enrichment analysis was accomplished with package ClusterProfiler (version 3.7) in R (version 3.4.4) (Yu et al. 2012).

### Immunoblotting Analysis

The total proteins were extracted from the flag leaves of 4-week-old seedling grown under control light. Immunoblotting analysis was performed according to a previous study (Wang and Blumwald 2014) with anti-Actin antibodies (Agrisera, AS132640) or anti-FLAG antibodies (Sigma, A8592-2MG).

## Supplementary information

**Additional file 1: Table S1.** The related information of all the peptides identified in proteomic analysis.

**Additional file 2: Table S2.** Protein groups and majority proteins identified in proteomic analysis.

**Additional file 3: Table S3.** Differentially expressed proteins between control light (0-NIP-CK) and low light (1-NIP-LL).

**Additional file 4: Table S4.** GO enrichment for all DEPs in cellular components.

**Additional file 5: Table S5.** KEGG enrichment for all DEPs.

**Additional file 6: Table S6.** The primers used in the construction of *OsGAPB* overexpression.

## Data Availability

All data generated or analyzed during this study are included in this published article and its supplementary information files.

## Abbreviations

iTRAQ: Isobaric tags for relative and absolute quantitation
DEP: Differentially-expressed protein
OsGAPB: β subunit of glyceraldehyde-3-phosphate dehydrogenase
Rubisco: Ribulose-1,5-bisphosphate carboxylase/oxygenase
ETR: Electron transport
Cyt: Cytochrome
PS: Photosystem
NIP: Nippobare
CL: Control light
LL: Low light
MS/MS: Tandem mass spectrometry
FC: Fold change
GO: Gene ontology
KEGG: Kyoto encyclopedia of genes and genomes
LHCA and LHCB: Light-harvesting complex A subunit and B subunit
GAPDH: Glyceraldehyde-3-phosphate dehydrogenase
OE: Overexpression
D1/PsbA: Subunit A of photosystem II
PRK: Phosphoribulokinase

## References

[CR1] Abbasi FM, Komatsu S (2004). A proteomic approach to analyze salt-responsive proteins in rice leaf sheath. Proteomics.

[CR2] Agarwal GK, Rakwal R, Yonekura M, Kubo A, Saji H (2002). Proteome analysis of differentially displayed proteins as a tool for investigating ozone stress in rice (Oryza sativa L.) seedlings. Proteomics.

[CR3] Baalmann E, Scheibe R, Cerff R, Martin W (1996). Functional studies of chloroplast glyceraldehyde-3-phosphate dehydrogenase subunits A and B expressed in Escherichia coli: formation of highly active A4 and B4 homotetramers and evidence that aggregation of the B4 complex is mediated by the B subunit carboxy terminus. Plant Mol Biol.

[CR4] Bi HG, Dong XB, Wu GX, Wang ML, Ai XZ (2015). Decreased TK activity alters growth, yield and tolerance to low temperature and low light intensity in transgenic cucumber plants. Plant Cell Rep.

[CR5] Bi HG, Liu PP, Jiang ZS, Ai XZ (2017). Overexpression of the rubisco activase gene improves growth and low temperature and weak light tolerance in Cucumis sativus. Physiol Plant.

[CR6] Buchanan BB, Balmer Y (2005). Redox regulation: a broadening horizon. Annu Rev Plant Biol.

[CR7] Cao YM, Zhang ZQ, Zhang T, You Z, Geng JC, Wang YF, Hu TM, Yang PZ (2018). Overexpression of zeaxanthin epoxidase gene from Medicago sativa enhances the tolerance to low light in transgenic tobacco. Acta Biochim Pol.

[CR8] Chang LL, Guo AP, Jin X, Yang Q, Wang D, Sun Y, Huang QX, Wang LM, Peng CZ, Wang XC (2015). The beta subunit of glyceraldehyde 3-phosphate dehydrogenase is an important factor for maintaining photosynthesis and plant development under salt stress-based on an integrative analysis of the structural, physiological and proteomic changes in chloroplasts in Thellungiella halophila. Plant Sci.

[CR9] Cui LR, Zou ZR, Zhang J, Zhao YY, Yan F (2016). 24-Epibrassinoslide enhances plant tolerance to stress from low temperatures and poor light intensities in tomato (Lycopersicon esculentum Mill.). Funct Integr Genomic.

[CR10] Cui SX, Huang F, Wang J, Ma X, Cheng YS, Liu JY (2005). A proteomic analysis of cold stress responses in rice seedlings. Proteomics.

[CR11] Du HM, Liang Y, Pei KQ, Ma KP (2011). UV radiation-responsive proteins in Rice leaves: a proteomic analysis. Plant Cell Physiol.

[CR12] Fermani S, Sparla F, Falini G, Martelli PL, Casadio R, Pupillo P, Ripamonti A, Trost P (2007). Molecular mechanism of thioredoxin regulation in photosynthetic A2B2-glyceraldehyde-3-phosphate dehydrogenase. Proc Natl Acad Sci U S A.

[CR13] Hajduch M, Rakwal R, Agrawal GK, Yonekura M, Pretova A (2001). High-resolution two-dimensional electrophoresis separation of proteins from metal-stressed rice (Oryza sativa L.) leaves: drastic reductions/fragmentation of ribulose-1,5-bisphosphate carboxylase/oxygenase and induction of stress-related proteins. Electrophoresis.

[CR14] Howard TP, Metodiev M, Lloyd JC, Raines CA (2008). Thioredoxin-mediated reversible dissociation of a stromal multiprotein complex in response to changes in light availability. Proc Natl Acad Sci U S A.

[CR15] Hu L, Liao W, Dawuda MM, Yu J, Lv J (2017). Appropriate NH4(+): NO3(−) ratio improves low light tolerance of mini Chinese cabbage seedlings. BMC Plant Biol.

[CR16] Kaiser E, Morales A, Harbinson J (2018). Fluctuating light takes crop photosynthesis on a rollercoaster ride. Plant Physiol.

[CR17] Keren N, Berg A, VanKan PJM, Levanon H, Ohad I (1997). Mechanism of photosystem II photoinactivation and D1 protein degradation at low light: the role of back electron flow. P Natl Acad Sci USA.

[CR18] Lee DG, Ahsan N, Lee SH, Kang KY, Bahk JD, Lee IJ, Lee BH (2007). A proteomic approach in analyzing heat-responsive proteins in rice leaves. Proteomics.

[CR19] Leong TY, Anderson JM (1984). Adaptation of the thylakoid membranes of pea chloroplasts to light intensities. II. Regulation of electron transport capacities, electron carriers, coupling factor (CF1) activity and rates of photosynthesis. Photosynth Res.

[CR20] Liu J, Xu M, Estavillo GM, Delhaize E, White RG, Zhou M, Ryan PR (2018). Altered expression of the malate-permeable anion channel OsALMT4 reduces the growth of rice under low radiance. Front Plant Sci.

[CR21] Liu QH, Wu X, Chen BC, Ma JQ, Gao J (2014). Effects of low light on agronomic and physiological characteristics of rice including grain yield and quality. Rice Sci.

[CR22] Liu QH, Zhou XB, Yang LQ, Li T, Zhang JJ (2009). Effects of early growth stage shading on rice flag leaf physiological characters and grain growth at grain-filling stage. Ying Yong Sheng Tai Xue Bao.

[CR23] Liu W, Su J (2016). Effects of light acclimation on shoot morphology, structure, and biomass allocation of two Taxus species in southwestern China. Sci Rep.

[CR24] Lu T, Yu HJ, Li Q, Chai L, Jiang WJ (2019). Improving plant growth and alleviating photosynthetic inhibition and oxidative stress from low-light stress with exogenous GR24 in tomato (Solanum lycopersicum L.) seedlings. Front Plant Sci.

[CR25] Mullineaux CW, Emlyn-Jones D (2005). State transitions: an example of acclimation to low-light stress. J Exp Bot.

[CR26] Nayak SK, Murty KS (1980). Effect of varying light intensities on yield and growth-parameters in rice. Indian J Plant Physiol.

[CR27] Qu MN, Zheng GY, Hamdani S, Essemine J, Song QF, Wang HR, Chu CC, Sirault X, Zhu XG (2017). Leaf photosynthetic parameters related to biomass accumulation in a global rice diversity survey. Plant Physiol.

[CR28] Ruberti I, Sessa G, Ciolfi A, Possenti M, Carabelli M, Morelli G (2012). Plant adaptation to dynamically changing environment: the shade avoidance response. Biotechnol Adv.

[CR29] Salekdeh GH, Siopongco J, Wade LJ, Ghareyazie B, Bennett J (2002). Proteomic analysis of rice leaves during drought stress and recovery. Proteomics.

[CR30] Sekhar S, Panda D, Kumar J, Mohanty N, Biswal M, Baig MJ, Kumar A, Umakanta N, Samantaray S, Pradhan SK, Shaw BP, Swain P, Behera L (2019). Comparative transcriptome profiling of low light tolerant and sensitive rice varieties induced by low light stress at active tillering stage. Sci Rep.

[CR31] Singh R, Jwa NS (2013). Understanding the responses of rice to environmental stress using proteomics. J Proteome Res.

[CR32] Sun YY, Sun YJ, Chen L, Xu H, Ma J (2012). Effects of different sowing dates and low-light stress at heading stage on the physiological characteristics and grain yield of hybrid rice. Ying Yong Sheng Tai Xue Bao.

[CR33] Tian Y, Sacharz J, Ware MA, Zhang H, Ruban AV (2017). Effects of periodic photoinhibitory light exposure on physiology and productivity of Arabidopsis plants grown under low light. J Exp Bot.

[CR34] Tikkanen M, Pippo M, Suorsa M, Sirpio S, Mulo P, Vainonen J, Vener A, Allahverdiyeva Y, Aro EM (2006). State transitions revisited - a buffering system for dynamic low light acclimation of Arabidopsis. Plant Mol Biol.

[CR35] Trost P, Fermani S, Marri L, Zaffagnini M, Falini G, Scagliarini S, Pupillo P, Sparla F (2006). Thioredoxin-dependent regulation of photosynthetic glyceraldehyde-3-phosphate dehydrogenase: autonomous vs. CP12-dependent mechanisms. Photosynth Res.

[CR36] Venkateswarlu B (1977). Influence of low light-intensity on growth and productivity of rice, Oryza-Sativa-L. Plant Soil.

[CR37] Vialet-Chabrand S, Matthews JS, Simkin AJ, Raines CA, Lawson T (2017). Importance of fluctuations in light on plant photosynthetic acclimation. Plant Physiol.

[CR38] Viji MM, Thangaraj M, Jayapragasam M (1997). Low irradiance stress tolerance in rice (Oryza sativa L). Biol Plant.

[CR39] von Caemmerer S, Farquhar GD (1981). Some relationships between the biochemistry of photosynthesis and the gas exchange of leaves. Planta.

[CR40] Wang L, Deng F, Ren WJ, Yang WY (2013). Effects of shading on starch pasting characteristics of indica hybrid rice (Oryza sativa L.). PLoS One.

[CR41] Wang SH, Blumwald E (2014). Stress-induced chloroplast degradation in Arabidopsis is regulated via a process independent of autophagy and senescence-associated vacuoles. Plant Cell.

[CR42] Xiong Q, Zhong L, Shen T, Cao C, He H, Chen X (2019). iTRAQ-based quantitative proteomic and physiological analysis of the response to N deficiency and the compensation effect in rice. BMC Genomics.

[CR43] Yu GC, Wang LG, Han YY, He QY (2012). clusterProfiler: an R package for comparing biological themes among gene clusters. Omics.

[CR44] Zhang J, Tang W, Huang Y, Niu X, Zhao Y, Han Y, Liu Y (2015). Down-regulation of a LBD-like gene, OsIG1, leads to occurrence of unusual double ovules and developmental abnormalities of various floral organs and megagametophyte in rice. J Exp Bot.

[CR45] Zivcak M, Brestic M, Kalaji HM, Govindjee (2014). Photosynthetic responses of sun- and shade-grown barley leaves to high light: is the lower PSII connectivity in shade leaves associated with protection against excess of light?. Photosynth Res.

